# How well can poor child health and development be predicted by data collected in early childhood?

**DOI:** 10.1136/jech-2018-211028

**Published:** 2018-09-21

**Authors:** Viviane S Straatmann, Anna Pearce, Steven Hope, Benjamin Barr, Margaret Whitehead, Catherine Law, David Taylor-Robinson

**Affiliations:** 1 Department of Public Health and Policy, University of Liverpool, Liverpool, UK; 2 MRC/CSO Social and Public Health Sciences Unit, Institute of Health and Wellbeing, University of Glasgow, Glasgow, UK; 3 Population, Policy and Practice, UCL Great Ormond Street Institute of Child Health, London, UK

**Keywords:** child health, health inequalities, public health policy, social epidemiology, health services

## Abstract

**Background:**

Identifying children at risk of poor developmental outcomes remains a challenge, but is important for better targeting children who may benefit from additional support. We explored whether data routinely collected in early life predict which children will have language disability, overweight/obesity or behavioural problems in later childhood.

**Methods:**

We used data on 10 262 children from the UK Millennium Cohort Study (MCS) collected at 9 months, 3, and 11 years old. Outcomes assessed at age 11 years were language disability, overweight/obesity and socioemotional behavioural problems. We compared the discriminatory capacity of three models: (1) using data currently routinely collected around the time of birth; (2) Model 1 with additional data routinely collected at 3 years; (3) a statistically selected model developed using a larger set of early year’s risk factors for later child health outcomes, available in the MCS—but not all routinely collected.

**Results:**

At age 11, 6.7% of children had language disability, 26.9% overweight/obesity and 8.2% socioemotional behavioural problems. Model discrimination for language disability was moderate in all three models (area under the curve receiver-operator characteristic 0.71, 0.74 and 0.76, respectively). For overweight/obesity, it was poor in model 1 (0.66) and moderate for model 2 (0.73) and model 3 (0.73). Socioemotional behavioural problems were also identified with moderate discrimination in all models (0.71; 0.77; 0.79, respectively).

**Conclusion:**

Language disability, socioemotional behavioural problems and overweight/obesity in UK children aged 11 years are common and can be predicted with moderate discrimination using data routinely collected in the first 3 years of life.

## Background

The antenatal period and first 2–3 years are crucial stages that influence children’s subsequent development and health outcomes. By age 3 years, many physical, cognitive and emotional development problems are apparent, but there remain opportunities to intervene to improve child outcomes.^1–3^ There is increasing recognition of the need to collect better early years’ data to identify children most at risk early, in order to facilitate more appropriate referral to services and early intervention programmes.^4^ Accordingly, the National Health Service (NHS) in England has been developing an improved national maternity services dataset, to collate routinely collected sociodemographic and perinatal information. In addition, in 2015, a new ‘integrated universal health check’ was introduced for children aged 2–3 years in England to provide a more complete picture of children’s health and development.^3–5^

A central challenge in using these new datasets is to accurately identify children most in need of additional support to achieve their greater long-term health and developmental potential and then deciding the most appropriate combination of universal and targeted service.^6 7^ Predictive risk models, used widely for applications such as cardiovascular risk prediction,^8–10^ have not been extensively assessed to inform child public health interventions. One previous study using a UK cohort showed that maternal age was a poor predictor of child health and development up to age 5 and that prediction was improved by including data on mother’s smoking status during pregnancy, education level, mental health and financial status.^11^ An Australian study using linked early childhood data to identify children with poor development at school entry showed that a model with six perinatal predictors (maternal age, smoking, parity, marital status and both parents’ occupation) demonstrated similar discrimination to a model including 22 predictors, constituting a more statistically parsimonious set of perinatal characteristics for predicting developmental vulnerability.^12^

In the context of the new datasets being collected in England, the aim of this study was to explore how early childhood characteristics predict three important developmental outcomes: language disabilities (cognitive outcome), overweight/obesity (physical outcome) and socioemotional behavioural problems (behavioural outcome) in later childhood (11 years). To address this aim, we used data from the UK Millennium Cohort Study (MCS), a nationally representative study of infants born in the early 2000s in the UK, which provides a rich data source on the social context and measures of health for children growing up in the UK.^13^ We assess the predictive capacity of a model using data routinely collected in maternity services; determine how the model’s performance improves when this is updated with information collected at age 2–3 years and compare the performance of the enhanced model with a third model using a larger range of early life risk factors for adverse child health collected in MCS.

## Methods

### Data source and study population

The MCS is a nationally representative sample of children born in the UK between September 2000 and January 2002 and followed up at intervals (sweeps) to the present date. We chose the MCS as it captures a wide range of data on the social context for children growing up in the UK and provides actual measures of both early and late child health outcomes that can be used to develop predictive models.

The MCS study oversampled children living in disadvantaged areas and those with high proportions of ethnic minority groups, and non-response weights were used to address sample attrition. Further information on the cohort and sampling design can be found in the cohort profile.^13^ Interviews were carried out by trained interviewers in the home with the main respondent (usually the mother). We used data from three sweeps when the children were aged 9 months, 3 years and 11 years. Information was collected from 18 818 infants (91% of the 20 646 in the target sample), and analysis was restricted to 18 296 singleton children.

### Cognitive, physical and behavioural outcomes

We investigated outcomes at 11 years old, an important transition stage between childhood and adolescence marked by the end of primary school. Cognitive ability was evaluated through the British Ability Scale Second Edition (BAS II) Verbal Similarities test, a validated standardised assessment of verbal reasoning and knowledge, normed for children and adolescents from 3 years to 11 years of age.^14 15^ We defined children as having language disability if they scored –1.25 SD below the normed mean score for the sample.^16–18^ Overweight/obesity was derived from the body mass index (BMI), using the age and sex-specific International Obesity Task Force cut-offs.^19^ The Strengths and Difficulties Questionnaire (SDQ—maternal report) was used to assess child socioemotional behaviour. The SDQ is a 25 item measure that asks parents to rate their child’s behaviour over the previous 6 months using five subscales: peer problems, conduct disorders, hyperactivity, emotional problems and prosocial behaviour.^20^ As in previous studies,^21 22^ we used the total difficulties score (excluding the prosocial behaviour subscale), dichotomised at the validated ‘borderline-abnormal’ (17–40), cut-off score, indicating socioemotional behavioural problems.^20^

### Potential predictors

**Figure 1:**
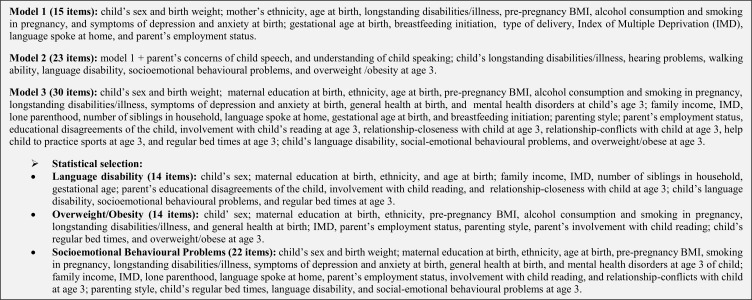
Description of items included in models 1, 2 and 3.

We outline predictors used in this study, grouped as perinatal (MCS first sweep-9 months), age 3 years (MCS second sweep) and earlier measures of language, SDQ and BMI at age 3 (figure 1). The full details of the coding of the predictors are provided in the online supplementary material.

10.1136/jech-2018-211028.supp1 ementary file 1

### Modelling approach

We developed three models:

Model 1: using variables in the MCS that are also currently collected routinely around the time of birth in maternity services in England (15 items). These data have been collected in the NHS in England and collated in the Maternity Services Data Set from April 2015 onwards.^23^

Model 2: using variables collected in maternity services (model 1) plus additional information collected at age 3 years in MCS which capture the five central domains included in the new integrated 2.5-year-old health check in England: (1) personal, social and emotional development, (2) communication and language, (3) physical health, (4) learning and cognitive development and (v) physical development and self-care)^2^ (23 items (15 items of model 1 plus 8 items)).

Model 3: a model including 30 perinatal, family/environmental and early childhood factors up to age 3 years, selected from risk factors for later child health and development problems identified in studies worldwide.^24–29^ Items included in this model overlap all items of model 1 and 18 items of model 2, since we did not include all variables capturing domains of the Ages and Stages Questionnaire (ASQ) which are represented by other instruments.

We applied a statistical selection to the saturated model (30 items), and a predictive model was developed based on statistical parsimony for each outcome. Figure 1 shows the complete description of items included in each model.

### Statistical analyses

First, we assessed the prevalence (%) for all potential predictor and outcome variables. Relative risks (RRs) and 95% CI for outcomes at 11 years were estimated using Poisson regression for all predictors included in models 1, 2 and 3. To develop model 3, we began with a saturated model containing the full range of 30 variables listed above and then selected a smaller number of variables using forwards and backwards stepwise selection (p≤0.1 for inclusion and p>0.11 for exclusion). Sampling and response weights were not used for receiver-operator characteristic (ROC) analysis.

The predicted probability of poor child development was calculated from these regression models. Predictive risk modelling was performed using a ROC curve which is a graphical plot that illustrates the diagnostic ability of a binary classifier system as its discrimination of true positives (ie, sensitivity) versus the fraction of false positives (ie, 1-specificity).^30^ For each model, we assessed the probability cut-off point to obtain the optimal maximised probability cut-off using a function of the difference between true positive rate and false positive rate over all possible cut-point values. The optimal maximised cut-off is the point where the sensitivity and specificity curves intersected and classifies most of the individuals correctly.^31^ Area under the receiver operating characteristic curve (AUROC) indicates the model’s overall capacity to discriminate between those who have or do not have the outcome. This provides an indication of how well the models perform in terms of the probability that a random pair of one child with the poor outcome and one without would be correctly ranked by the predicted probabilities from the model. A guide for classifying the accuracy of a diagnostic test is AUC values of ‘0.90–1=excellent’, ‘0.80–0.90=good’, ‘0.70–0.80=moderate’, ‘0.60–0.70=poor’ and ‘0.50–0.60=fail’.^31 32^

The integrated discrimination improvement (IDI) for model 2 compared with model 1 and model 3 with model 2 were also calculated. The IDI assesses discrimination without relying on cut-off points and compares the average difference in predicted risk for children with poor health or development with those which do not have poor health or development. The IDI improvement is greater when the second model correctly assigns individuals to higher or lower probabilities of having the outcome in comparison to the first model.^33^ Calibration’s accuracy of the models was assessed using the Hosmer-Lemeshow goodness-of-fit χ² statistic. In this statistic test, the null hypothesis is that predicted proportion equals the observed proportion within ranked groupings (deciles) of predicted risk and a high p value suggests good calibration of predicted and observed risk.^34^ Dominance analysis, a method for assessing the relative weight of predictive variables in a multivariable regression, was used to estimate the standardised dominance score (SDS) to rank the importance of each variable in each model.^35^ All analyses were conducted in Stata SE V.13.0 (Stata, 2014).

### Multiple imputation

Multiple imputation by chained equation was performed to impute missing data using the ‘mi impute chained’ command in Stata SE V.13.0 (Stata, 2014). We used data of predictors and the three outcomes at age 11 to shape the imputation process of the other risk factors included in the three models above (imputed sample, n=10 262). We generated 20 datasets, with 200 iterations per imputed dataset. Results were calculated by averaging the results across the 20 imputed datasets using Rubin’s rules.^36^ Results from the imputed sample are reported below and for the complete case sample are provided in the supplementary material.

## Results

**Figure 2:**
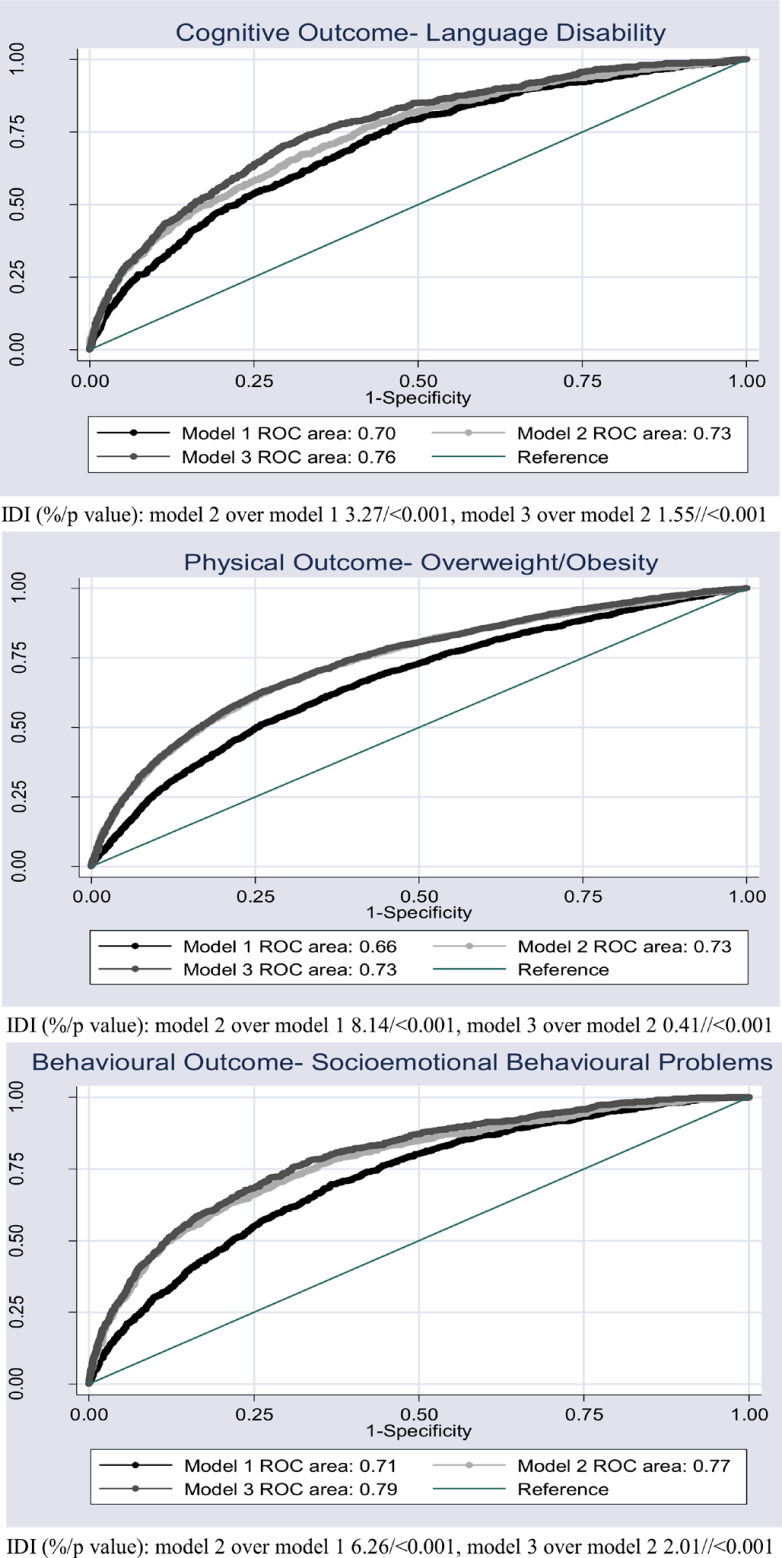
AUC and IDI of language disability, overweight/obesity and socioemotional behaviour problems at age 11 for UK children. AUC, area under the curve; IDI, integrated discrimination improvement; ROC, receiver-operator characteristic.

At 11 years, 6.7% (95% CI 6.3% to 7.2%) of children had language disability; 26.9% (95% CI 26.1% to 27.8%) overweight/obesity and 8.2% (95% CI 7.6% to 8.7%) had socioemotional behavioural problems. Prevalence of outcomes stratified by risk factors is shown in the online supplementary material. With regard to the statistical selection method applied to develop model 3, the language disability and overweight/obesity models included 14 variables, and 22 variables were selected for the socioemotional behavioural problems model (figure 1). Figure 2 shows the ROC curve for each outcome in separate panels, with model 1 in black, model 2 in light grey and model 3 in dark grey: Language disability was identified with moderate discrimination ability for model 1 (AUROC: 0.70 95% CI 0.68 to 0.72), model 2 (AUROC: 0.73, 95% CI 0.71 to 0.75) and model 3 (AUROC: 0.76, 95% CI 0.74 to 0.78). Overweight/obesity was identified with poor discrimination in model 1 (AUROC: 0.66, 95% CI 0.65 to 0.67) and moderate discrimination for models 2 (AUROC: 0.73, 95% CI 0.72 to 0.74) and model 3 (AUROC: 0.73, 95% CI 0.72 to 0.74). Socioemotional behavioural problems were also identified with moderate discrimination in all models (model 1: AUROC: 0.71, 95% CI 0.69 to 0.73; model 2: AUROC: 0.77, 95% CI 0.75 to 0.79; model 3: AUROC: 0.79, 95% CI 0.77 to 0.80, respectively). IDI indicated that model 2 resulted in a significant improvement in discrimination over model 1, particularly for overweight/obesity and socioemotional behavioural problems with 8.14% and 6.26% more children being correctly reclassified by model 2 compared with model 1, respectively. The IDI improvement was smaller for model 3 compared with model 2 for all outcomes, but remained significant (figure 2).

Sensitivity, specificity, positive predictive value, negative predictive value, percentage of positives and correctly classified for all models are shown in table 1. Model 2 was the most accurate model for all outcomes, which means that this model had the best correct classification of children with health and development problems. Table 2 presents the multivariable associations between risk factors and language disability, overweight/obesity and socioemotional behavioural problems at age 11, calibration and dominance analyses for model 2 (model with the best correct classification of children for all outcomes). Those results for models 1 and 3 can be found in the online supplementary material.

**Table 1:** Test properties of maximised cut-off probability for language disability, overweight/obese and socioemotional behavioural problems at age 11

Test properties	Maximised cut-offs								
	Cognitive language disability (%)*			Physical overweight/obese (%)†			Behavioural socioemotional problems (%)‡		
	Model 1	Model 2	Model 3	Model 1	Model 2	Model 3	Model 1	Model 2	Model 3
Sensitivity	57.0	55.8	68.5	64.2	66.7	68.2	60.7	62.2	69.6
Specificity	71.6	77.4	71.62	60.7	69.1	67.6	70.3	79.3	74.0
PPV	12.7	15.2	14.89	37.6	44.3	43.7	15.4	21.0	19.2
NPV	95.8	96.0	96.9	82.2	84.9	85.2	95.3	95.9	96.5
% of positives	30.3	24.8	31.1	46.0	40.5	42.0	32.2	24.1	29.5
Correctly classified	70.6	75.9	71.4	61.7	68.5	67.8	69.5	77.9	73.7

Millennium Cohort Study, 2001–2012, UK (imputed data, n=10 262).

*Maximised cut-offs used for language disability (model 1: 0.08, model 2: 0.08, model 3: 0.07).

†Maximised cut-offs used for overweight/obese (model 1: 0.24, model 2: 0.26, model 3: 0.25).

‡Maximised cut-offs used for socioemotional behavioural problems (model 1: 0.09, model 2: 0.09, model 3: 0.08).

% of positives: total of children classified as positive, even if it is true or not; correctly classified: true positives plus true negatives; NPV, negative predictive value; PPV, positive predictive value.

**Table 2:** Multivariable associations between factors included in model 2 and language disability, overweight/obese and socioemotional behavioural problems at age 11

Model 2*						
Outcomes to age 11	Cognitive language disability		Physical overweight/obese		Behavioural socioemotional problems	
Predictors	Relative risk (95% CI)	SDS/Ranking†	Relative risk (95% CI)	SDS/Ranking†	Relative risk (95% CI)	SDS/Ranking†
Mother ethnicity		0.017/10		0.006/11		0.005/17
White	Ref		Ref		Ref	
Mixed	0.99 (0.50 to 1.95)		0.77 (0.53 to 1.10)		1.74 (0.96 to 3.15)	
Indian	0.37 (0.19 to 0.71)		1.25 (0.94 to 1.67)		0.86 (0.44 to 1.67)	
Pakistani	1.13 (0.70 to 1.85)		1.32 (1.04 to 1.68)		0.94 (0.54 to 1.63)	
Bangladeshi	1.64 (0.96 to 2.82)		1.12 (0.79 to 1.59)		0.98 (0.48 to 1.98)	
Black	0.73 (0.43 to 1.25)		1.34 (1.13 to 1.58)		1.23 (0.74 to 2.03)	
Other	0.61 (0.25 to 1.47)		0.81 (0.56 to 1.17)		0.36 (0.12 to 1.04)	
Mother’s age at birth		0.020/9		0.002/14		0.003/7
14–19 years old	1.16 (0.77 to 1.74)		0.87 (0.73 to 1.05)		1.37 (0.98 to 1.91)	
20–24 years old	1.27 (0.92 to 1.73)		0.93 (0.82 to 1.05)		1.48 (1.11 to 1.97)	
25–29 years old	1.02 (0.75 to 1.40)		0.90 (0.81 to 1.01)		1.26 (0.99 to 1.62)	
30–34 years old	1.05 (0.78 to 1.43)		0.97 (0.87 to 1.07)		1.01 (0.79 to 1.30)	
≥35 years old	Ref		Ref		Ref	
Language spoke at home		0.026/8		0.001/15		0.010/14
Only English	Ref		Ref		Ref	
English and additional language	0.64 (0.43 to 0.95)		0.98 (0.84 to 1.13)		0.70 (0.48 to 1.02)	
Not English	0.61 (0.39 to 0.95)		0.93 (0.72 to 1.21)		0.37 (0.18 to 0.75)	
Parents employment status		0.142/2		0.015/5		0.064/4
Both parents in work	Ref		Ref		Ref	
One parent in work	1.13 (0.90 to 1.51)		1.08 (0.90 to 1.12)		0.89 (0.91 to 1.54)	
Neither parent in work	1.98 (1.12 to 4.18)		1.32 (0.96 to 1.41)		1.93 (1.19 to 2.32)	
Deprivation-IMD		0.068/5		0.020/4		0.026/9
1 quintile- highest	Ref		Ref		Ref	
2 quintiles	1.45 (0.98 to 2.22)		1.13 (0.97 to 1.31)		0.84 (0.60 to 1.19)	
3 quintiles	1.66 (1.12 to 2.45)		1.14 (0.99 to 1.31)		1.03 (0.75 to 1.41)	
4 quintiles	1.57 (1.07 to 2.31)		1.15 (1.00 to 1.33)		1.40 (1.04 to 1.88)	
5 quintile- lowest	1.84 (1.28 to 2.66)		1.23 (1.06 to 1.42)		1.10 (0.81 to 1.49)	
Child gender		0.009/14		0.013/6		0.023/11
Male	Ref		Ref		Ref	
Female	1.30 (1.10 to 1.54)		1.15 (1.06 to 1.24)		0.71 (0.60 to 0.83)	
Child birth weight		0.008/16		0.001/17		0.001/20
Normal (≥2.5 to ≤4.5 kg)	Ref		Ref		Ref	
Low (<2.5 kg)	0.92 (0.65 to 1.30)		1.00 (0.84 to 1.20)		1.19 (0.88 to 1.62)	
High (>4.5 kg)	1.19 (0.71 to 1.99)		1.10 (0.83 to 1.34)		0.70 (0.33 to 1.48)	
Gestational age		0.001/22		0.001/16		0.001/23
Term, 37–41 weeks	Ref		Ref		Ref	
Preterm, 23 to 36 weeks	1.50 (1.03 to 2.17)		1.00 (0.85 to 1.19)		1.01 (0.70 to 1.45)	
Post-term, 42– 43 weeks	0.79 (0.59 to 1.05)		1.10 (1.00 to 1.22)		0.97 (0.78 to 1.21)	
Smoking in pregnancy		0.017/11		0.026/3		0.067/2
None	Ref		Ref		Ref	
1–10 cigarettes/day	1.01 (0.78 to 1.31)		1.15 (1.03 to 1.29)		1.18 (0.98 to 1.43)	
11–20 cigarettes/day	0.94 (0.63 to 1.41)		1.34 (1.14 to 1.57)		1.63 (1.24 to 2.13)	
>20 cigarettes/day	1.71 (1.04 to 2.28)		1.38 (1.04 to 1.83)		1.31 (0.83 to 2.05)	
Alcohol consumption in pregnancy		0.016/12		0.010/7		0.004/19
No	Ref		Ref		Ref	
Yes	0.94 (0.75 to 1.16)		0.88 (0.81 to 0.95)		0.95 (0.80 to 1.14)	
Breastfeeding initiation		0.029/7		0.008/8		0.004/18
Yes	Ref		Ref		Ref	
No	1.05 (0.87 to 1.27)		1.00 (0.91 to 1.10)		0.94 (0.81 to 1.10)	
Maternal depression or anxiety		0.009/15		0.007/9		0.071/6
No Yes	Ref 1.08 (0.90 to 1.30)		Ref 1.06 (0.98 to 1.16)		Ref 1.33 (1.13 to 1.57)	
Type of delivery		0.001/19		0.002/12		0.001/22
Normal Assisted (forceps, vacuum, breach)	Ref 1.17 (0.80 to 1.70)		Ref 0.90 (0.79 to 1.01)		Ref 1.00 (0.71 to 1.40)	
Planned caesarean	1.18 (0.88 to 1.58)		1.06 (0.93 to 1.20)		0.81 (0.63 to 1.04)	
Emergency caesarean	1.03 (0.80 to 1.34)		0.99 (0.89 to 1.11)		1.07 (0.86 to 1.32)	
Other	1.10 (0.26 to 4.61)		0.82 (0.47 to 1.43)		1.17 (0.57 to 2.37)	
Mother BMI before born		0.007/17		0.291/2		0.017/12
Normal	Ref		Ref		Ref	
Overweight/obese	1.26 (1.05 to 1.52)		1.85 (1.70 to 2.00)		1.32 (1.13 to 1.53)	
Mother disability or illness		0.004/21		0.006/10		0.025/10
No	Ref		Ref		Ref	
Yes	0.88 (0.72 to 1.07)		1.07 (0.98 to 1.17)		1.26 (1.08 to 1.46)	
Hearing problems age 3		0.008/20		0.001/23		0.007/16
No	Ref		Ref		Ref	
Yes	0.93 (0.64 to 1.36)		1.12 (0.97 to 1.29)		1.10 (0.83 to 1.46)	
Concern about child’s speech age 3		0.043/6		0.001/21		0.066/3
No	Ref		Ref		Ref	
Yes	1.60 (1.23 to 2.08)		1.01 (0.90 to 1.15)		1.59 (1.30 to 1.95)	
Understands child’s speech age 3		0.108/3		0.001/20		0.046/5
Always	Ref		Ref		Ref	
Sometimes	0.97 (0.64 to 1.47)		0.75 (0.57 to 0.98)		0.88 (0.60 to 1.30)	
Rarely	2.16 (1.35 to 3.46)		1.10 (0.68 to 1.76)		1.08 (0.63 to 1.85)	
Walkup steps age 3		0.011/13		0.002/13		0.010/15
Yes	Ref		Ref		Ref	
With help	1.00 (0.57 to 1.76)		0.79 (0.59 to 1.07)		1.07 (0.73 to 1.57)	
No	1.67 (1.08 to 2.58)		1.28 (1.00 to 1.63)		1.27 (0.86 to 1.88)	
Child disability or illness age 3		0.004/18		0.001/22		0.031/8
No	Ref		Ref		Ref	
Yes	1.09 (0.87 to 1.35)		0.96 (0.88 to 1.05)		1.36 (1.16 to 1.60)	
Naming vocabulary disability age 3		0.362/1		0.001/19		0.015/13
No language disability	Ref		Ref		Ref	
Language disability	2.51 (1.95 to 3.23)		0.98 (0.86 to 1.13)		1.10 (0.83 to 1.45)	
SDQ age 3		0.094/4		0.001/18		0.502/1
No related problems	Ref		Ref		Ref	
Behavioural problems	1.39 (1.12 to 1.72)		0.98 (0.87 to 1.10)		2.68 (2.22 to 3.23)	
BMI age 3		0.001/23		0.589/1		0.001/21
Normal weight	Ref		Ref		Ref	
Overweight/obese	0.92 (0.73 to 1.17)		2.47 (2.28 to 2.67)		1.05 (0.88 to 1.27)	
Hosmer-Lemeshow/p values‡	5.19/0.737		4.65/0.794		14.42/0.071	

MCS, 2001–2012, UK (imputed data, n=10 262).

*Model 2 includes information collected in maternity services in England plus correspondent factors assessed in MCS at age 3 that are collected on 2.5-year-old health check in England.

†SDS and weighted ranking of predictive risk variables.

‡Calibration analyses.

BMI, body mass index; IMD, Index of Multiple Deprivation; MCS, Millennium Cohort Study; SDQ, Strengths and Difficulties Questionnaire; SDS, Standardised Dominance Statistic.

The Hosmer-Lemeshow goodness-of-fit tests indicate adequate calibration in model 2 for all outcomes (Hosmer-Lemeshow/p value: language disability 5.19/0.737; overweight/obesity 4.65/0.794; socioemotional behavioural problems, model 2 14.42/0.071). Dominance analyses for model 2 showed that the top four most relevant factors for socioemotional behaviours at age 11 years were socioemotional behavioural problems at age 3 (0.502), smoking in pregnancy (0.067), parental concerns about child speech at age 3 (0.066) and neither parent in work (0.064). The most dominant factors for language disability at age 11 were naming and vocabulary disabilities at age 3 (0.362), neither parent in work (0.142), parental concerns about understanding of child speech at age 3 (0.108) and socioemotional behavioural problems at age 3 (0.094). For overweight/obesity at age 11, overweight/obesity at age 3 (0.589), maternal pre-pregnancy BMI indicating overweight/obese (0.291), smoking in pregnancy (0.026) and greater deprivation of area of residence (0.020) were the most important items.

Sensitivity analyses of AUROC not including prior measures of the outcome show similar findings to our main results, with moderate discrimination in models 2–3 for socioemotional behavioural and language problems, but lower discrimination for obesity/overweight (about 68% for models 2 and 3—see online supplementary material). In dominance analyses, when we remove prior measures of the relevant outcome, the second, third most influential variables and so on rise in the rank of importance (online supplementary material). Repeating the analysis including all of the variables from the ASQ in model 3 did not alter the model selection or change the results.

## Discussion

Using UK data from the MCS, we show that information collected in the first 3 years of life can be a potential tool to predict adverse health and developmental outcomes at age 11 with moderate accuracy. The discriminatory capacity of a model using data collected in maternity services in England is improved when updated with data routinely collected at 2–3 years (particularly earlier measures of the relevant outcomes), but addition of wider set of perinatal, family/environmental and early childhood factors up to age 3 years did not alter risk prediction.

The first 3 years of life provide a unique opportunity to intervene and improve child development and subsequent adult outcomes.^7^ There has been a raft of policies promoting the benefits of early intervention, but the research base to support effective targeting of these initiatives is still emerging. Child health policy recommendations in the UK apply the principles of proportionate universalism, with universal services provided for all families and, in addition, progressively more intensive support targeted at those with greater need.^6 7^ In a technical sense, we would like to be able to find a set of characteristics (eg, maternal, partner, child and community) that accurately identify those children most at risk for poorer developmental outcomes, to help plan improved services for their future development.

While it is true that relative concentrations of poorer outcomes are higher in disadvantaged populations, to our knowledge, there has been little systematic work examining the extent to which these outcomes are predicted by risk factors earlier in the life course. The existing studies that have investigated this and have similarly demonstrated the utility of using data collected at birth to predict poor child health outcomes. Chittleborough and colleagues^11^ used a prospective, regional birth cohort in England to explore the predictive value of maternal age, compared with a model using six predictors (mother <20 years, low maternal education, single parent, financial difficulties, depression, smoking in pregnancy) for child development outcomes up to age 5 years. Predictive capacity was improved in this study by including other data, but was still classified as poor (AUROC=0.67). The authors concluded that, even though maternal age is used to target early years child health programmes in many countries, these interventions will have little impact at a population level, since the majority of at risk children will miss out on intervention if young maternal age is the sole or main means of identifying eligibility for the programmes.^11^

A recent study from Australia used linked administrative perinatal datasets linked to data from the Australian Early Development Census to assess whether poor child development at age 5 could be predicted at a population level.^12^ A model with six perinatal characteristics (low maternal age, mother’s marital status (never married, widowed, divorced or separated), mother and father’s occupation (home duties, students, pensioners, unemployed), high number of previous pregnancies resulting in births≥20 weeks and smoking in second half of pregnancy) had poor discrimination for boys (AUROC=0.68) and moderate discrimination for girls (AUROC=0.72). The authors suggest that even with poor-moderate capacity of the models, if these six characteristics were used for targeting intensive support services and the programme targeted families with at least three of the six perinatal risk factors, approximately 10% of families in the population would be identified as needing an intensive intervention soon after birth.^12^ Building on these findings, our study shows that risk predictions were not substantially improved using a wider range of variables in the first 3 years of life and that these data also have moderate predictive value for outcomes at 11 years.

Socioeconomic factors and early measures of the relevant outcomes were the most important predictive indicators for child health and development at age 11 years. However, removing the early measure of the outcome from the analysis did not impact greatly on prediction, especially for language disabilities and socioemotional behavioural problems (as tested in our sensitivity analyses). Despite the high prevalence of overweight/obesity, it is to have been expected that predictive power for this outcome would be lower without age 3 years measurement, due to biological influences.^25^ Recent findings from predictive modelling studies in high-income countries, in the UK and Australia^37^ and in the USA^38^ corroborate the importance of social factors for later child health and development outcomes, even in high-income countries. Another study from Brazil (a middle-income country), using the 2004 Pelotas Birth Cohort, assessed a predictive model of early life factors for a cognitive outcome (low IQ) at age 6 years. Twelve risk factors were included in the final model and dominance analyses showed that social factors were the most important predictors.^39^

A strength of our study is the use of a large, contemporary UK cohort. A wide range of information is collected in the MCS, which allowed us to explore a large set of demographic, perinatal and early childhood risk factors. Measured BMI, validated assessments of language disability and socioemotional behavioural problems in children were also advantages. The MCS thus allowed us to consider what might be achieved through linkage of administrative datasets in the UK and to assess what added predictive value extra data collection might provide.

A limitation of our study is the lack of an external validation sample. In addition, missing data and attrition are common to all cohort studies, but the similar results in complete case and imputed datasets in our study offer reassurance that the risk of bias is minimised. We note that model 2 in our analysis included early measures of the prior problem, and it could be the case that much of the predictive value in the model could be explained by these early measures. However, repeating the analysis without these measures suggests that this is not the case (online supplementary material). We based most of our results on maternal self-reported data and decisions were made around categorising prediction variables. We have used cohort data from the nationally representative MCS and we expect that the predictors identified in the MCS would predict outcomes similarly in the general population. However, it is unclear the extent to which these models can be reproduced in routinely collected data. Further limitations include concerns about how similar the measures in the MCS are to those used in health services, since MCS data variables are aimed at research and to capture a picture of a representative sample of all UK children. Furthermore, we do not have detailed data on any intervention or specialised services that children may have accessed, that may have attenuated the associations in our study.

Further research is needed to assess the utility and impact of predictive risk models for child health and development outcomes in routine practice. We have used cohort data from the nationally representative MCS, but it is unclear the extent to which these models can be reproduced in routinely collected data. While many of the variables used in model 1 in our analysis should be available in routine data, other variables such as breastfeeding status and early measures of maternal mental health are more difficult to capture and may be of poor quality in routine data collection systems. Furthermore, we require a better understanding of how predictive risk modelling tools could be used in the context of specific child health systems, for instance, in the UK, what proportion of children would go on to receive specialist intervention; what proportion of those would benefit from this and what would be the magnitude of any benefits.

In the UK and the USA, there have been some attempts to target services on the basis of child and family characteristics, and our study provides evidence as to which variables are likely to be useful for this purpose in clinical and public health practice.^40 41^ As many high-income countries collect these sort of data, it would be instructive to test how well they predict the same outcomes. The use of such tools raises ethical issues, for instance being labelled high risk could be stigmatising and any population level targeting approach would generate false positives (and false negatives), that would have opportunity costs for services locally. The implementation of risk prediction tools to guide policies would have to be carefully considered to ensure families were appropriately counselled and supported.

## Conclusion

New child health datasets have been developed in England, but it remains a challenge to harness these population-level administrative datasets to improve outcomes for children. Our analysis shows that language disability, socioemotional behavioural problems and overweight/obesity in UK children aged 11 years can be predicted with moderate discrimination using data routinely collected in England. Addition of further variables identified in the literature that mostly are not routinely collect in health services does not add considerable improvement on discriminatory capacity of health and development problems in later childhood. Further research is needed to identify what could increase the predictive power of these models at these and other ages in population-based databases such as MCS as well as assess how the dynamics of predictive algorithm models can be used in health services to identify children more likely to benefit from additional early years support.

**What is already known on this subject**

- Early identification of children at risk of poor health and developmental outcomes is challenging, and some existing studies suggest that data routinely collected in health services could be better used for this purpose.

**What this study adds**

- We show that language disability, socioemotional behavioural problems and overweight/obesity are common in UK children aged 11 years and can be predicted with moderate discrimination using data routinely collected in the first 3 years of life.
