# Supplementary material for: How well can poor child health and development be predicted by data collected in early childhood?
Source: J Epidemiol Community Health. 2018 Sep 21;72(12):1132–40. doi: 10.1136/jech-2018-211028 (PMC6252371; doi:10.1136/jech-2018-211028)

Supplementary material

Methods -Additional details

Overweight/obesity outcome: Height and weight data were collected by trained interviewers. Children were weighted with Tanita HD- 305 scales (Tanita UK Ltd., Middlesex, UK), without shoes or outdoor clothing; weights were recorded in kilograms to one decimal place. Heights were measured with the Leicester Height Measure Stadiometer (Seca Ltd., Birmingham, UK) and recorded to the nearest millimetre (Gray et al., 2010). Being overweight or obese was defined using the age and sex specific International Obesity Task Force (IOTF) cut-offs (Cole &Lobstein, 2012).

Gray J, Gatenby R, Simmonds N, Huang Y. Millenium Cohort Study Sweep 4 Technical Report 2nd edition. London: Centre for Longitudinal Studies (CLS); 2010.

*Cole TJ, Lobstein T. Extended international (IOTF) body mass index cut-offs for thinness, overweight and obesity. Pediatr Obes 2012; **7**: 284–294.*

Variables coding

Perinatal predictors

Child sex was coded as ‘male’ and ‘female’. Maternal ethnicity was classified as ‘white’, ‘mixed’, ‘Indian’, ‘Pakistani’, ‘Bangladeshi’, ‘Black’ and ‘other’. Maternal age at cohort child birth was measured in years, and categorised as ‘14-19’, ‘20-24’, ‘25-29’, ‘30-34’ and ‘over than 35’ years. Maternal education was categorized into six groups: (1) degree plus (higher degree and first degree qualifications), (2) diploma (in higher education), (3) A-levels, (4) grades A–C, (5) General Certificate of Secondary Education (GCSE) grades D–G, and (6) none of these qualifications. Parent’s employment status was classified into (1) both parents in work, (2) one parent in work, (3) neither parents in work. Language spoke at home was coded as ‘only English’, ‘English and additional language’ and ‘not English’. Poverty was defined as household equivalised income of less than 60% of national median household income equivalised according to the Organisation for Economic Co-operation and Development Household Equivalence Scale. Lone parenthood was defined was ‘lone’ vs. ‘not lone’. Number of siblings in household was coded as ‘1-2’ vs. ‘3 or more’. The Index of Multiple Deprivation (IMD) was used to assess area deprivation, based on the *Super Output Area* of the child’s postcode and divided into national quintiles.

Child weight at birth was classified as low (<2.5 kg), normal (2.5 to 4.5 Kg) or high (>4.5 kg). Gestational age was defined as preterm 26-36 weeks, term 37-41 weeks and post term 42-43 weeks. Breastfeeding initiation was coded as “had breastfeeding initiation” vs. “did not initiate breastfeeding”. Mode of delivery was classified as ‘normal’, ‘assisted- forceps, vacuum, breach’, ‘planned caesarean’, ‘emergency caesarean’ and ‘other’. Pre-pregnancy Body mass index (BMI) of mothers was categorized as ‘normal’ vs. ‘overweight/obese’. Smoking status in pregnancy was classified as ‘none’ ‘1-10 cigarettes per day’, ‘11-20 cigarettes per day’ and ‘>20 cigarettes’ per day’. Alcohol consumption in pregnancy was defined as ‘no’ vs. ‘yes’. Information on maternal general health was self-reported and defined as ‘excellent’, ‘good’, ‘fair’ or ‘poor’. Self-reported data of maternal symptoms of depression and anxious (ever have been diagnosed by doctor and treated) was coded as ‘had depression and anxious’ vs. ‘did not have depression and anxious’. Self-reported information about maternal longstanding disabilities or illness were coded as ‘yes-presence of disability’ vs. ‘no, absence of disabilities’.

Age 3 years predictors

Maternal mental health was assessed through the Kessler 6 scale to identify psychological distress; validated cut offs were used, contrasting normal (0–5) and moderate (6–24) distress scores (Kessler et al., 2002). Parental concerns about understanding of child speaking were classified as 'always', 'sometimes' and 'rarely'. Further concerns about hearing and speech were classified as 'some concerns' or 'none/not applicable'. The child ability to walk up steps was classified as 'yes-able alone', 'not able alone' and 'not able'. Mothers reported data about child longstanding disabilities or limiting illness, coded as 'yes-' vs. 'no'.

Parent's disagreement about child education was classified as 'none', 'less than once a week', 'once a week', 'several times a week' and 'at least once a day'. Parenting style was classified as 'firm rules and discipline', 'lots of fun', 'have not really thought about it', 'firm discipline plus lots of fun' and 'doing my best for the child'. Relationship of parents-child was assessed through the Pianta Child-Parent Relationship Scale (Pianta, 1995). Primary carer closeness was evaluated by seven items related to the level of warmth in the relationship with the child, which were summed and categorised as '35-highest warmth' '33-34', '30-32' and '7-29 -lowest warmth'[40]. Conflict scale was categorised into '7-15- lowest conflict', '16-20', '21-26' and '27-35- highest conflict'[40]. Involvement of parents to help the child to practice sports was classified as 'participative' vs. 'not participative, and to read was classified as 'every day', 'several times a week', 'once or twice a week', 'once or twice a month', 'less often and not at all'. Regular bed time was categorized as 'always', 'usually', 'sometimes', 'never'.

Kessler RC, Andrews G, Colpe L, et al. Short screening scales to monitor population prevalences and trends in non-specific psychological distress. Psychol Med 2002; 32: 959–76.

Pianta RC. Child-Parent Relationship Scale. Charlottesville: University of Virginia, 1995.

Results

Prevalence of the outcomes at age 11, stratified by the risk factors. Millennium Cohort Study, 2001-2012, United Kingdom (imputed data, N=10,262)

	Prevalence (%) N=10,232	Language disability (%) n= 693	Overweight / Obesity (%) n= 2,763	Socioemotional problems (%) n= 835
**Perinatal Predictors**				
**Mother ethnicity**				
White	88.1	6.1	26.3	8.2
Mixed	0.8	10.3	25.6	16.7
Indian	2.1	3.3	24.7	6.5
Pakistani	3.6	20.1	33.2	8.8
Bangladeshi	1.6	19.1	28.4	6.2
Black	2.5	5.9	41.4	8.2
Other	1.3	6.6	22.6	3.6
**Mother's age at birth**				
14-19 years old	6.7	9.9	25.4	13.6
20-24 years old	16.6	10.6	29.2	12.7
25-29 years old	27.8	6.4	27.3	8.4
30-34 years old	31.4	5.2	26.0	5.7
35-63 years old	17.5	5.4	26.4	5.9
**Language spoke at home**				
Only English	88.7	6.1	26.6	8.3
English and additional language	8.6	10.9	29.7	7.3
Not English	2.6	14.5	27.9	5.6
**Maternal education**				
Degree plus	19.9	1.6	18.5	2.8
Diploma	9.7	3.6	25.6	5.9
A-levels	10.6	3.5	23.8	6.0
GCSE Grades A-C	34.5	6.6	30.0	8.8
GCSE Grades D-G	10.5	11.9	31.7	13.0
None of these qualifications	14.8	14.7	30.8	13.4
**Number of siblings**				
1-2	77.6	5.7	26.3	7.7
3 or more	22.4	10.5	29.1	9.7
**Lone parenthood**				
No	86.5	6.2	26.2	7.1

Yes	13.5	10.6	31.4	15.3
**Parents employment status**				
Both parents in work	52.6	4.0	25.1	5.7
One parent in work	38.8	8.4	27.3	8.5
Neither parent in work	8.6	15.9	30.3	15.9
**Deprivation- IMD**				
1 quintile- highest	15.6	3.1	20.4	5.4
2 quintile	17.7	4.0	25.5	5.1
3 quintile	20.5	5.5	26.1	6.6
4 quintile	20.6	7.8	27.3	10.5
5 quintile- lowest	25.6	11.1	32.3	11.4
**Income**				
>60%	69.9	4.25	25.1	5.8
<60%	30.1	12.6	31.3	13.6
**Child gender**				
Male	50.4	6.4	24.9	9.8
Female	49.6	7.1	29.0	6.5
**Child birth weight**				
Normal (>=2.5 to <=4.5 kg)	92.1	6.5	26.9	7.9
Low (<2.5 kg)	6.0	11.0	25.0	11.9
High (>4.5 kg)	1.8	7.5	36.2	6.4
**Gestational age**				
Term, 37-41 weeks	82.8	6.5	26.7	8.1
Preterm, 23-36 weeks	5.7	11.2	26.5	10.5
Post-term, 42-43 weeks	11.5	6.1	28.9	7.5
**Smoking in pregnancy**				
None	79.2	6.1	25.2	6.5
1-10 cigarettes/day	15.9	8.4	32.7	12.9
11-20 cigarettes/day	3.9	10.1	36.3	18.8
> 20 cigarettes/day	0.9	17.6	39.6	20.9
**Alcohol consumption in pregnancy**				
No	68.1	7.7	28.7	8.8
Yes	31.9	4.8	23.2	6.9
**Breastfeeding initiation**				
Yes	70.0	5.6	25.3	7.2
No	30.0	9.4	30.8	10.4

**Maternal depression or anxiety**				
No	75.6	6.3	25.7	6.7
Yes	24.4	8.3	30.7	12.7
**Type of delivery**				
Normal	68.2	7.1	26.6	8.4
Assisted	9.3	5.0	22.8	7.6
Planned caesarean	9.3	6.2	31.3	6.8
Emergency caesarean	12.1	6.6	29.0	8.3
Other	0.4	4.7	27.9	14.0
**Mother BMI before born**				
Normal	70.5	6.3	20.4	7.3
Overweight/ obese	29.5	7.9	42.5	10.3
**Mother disability or illness**				
No	78.0	6.6	25.9	7.2
Yes	22.0	7.3	30.4	11.7
**Maternal general health**				
Excellent	31.0	5.0	23.1	4.9
Good	52.2	7.0	27.1	7.9
Fair	13.8	9.3	32.7	14.0
Poor	2.6	9.7	37.7	21.3
Age 3 Predictors				
**Maternal mental health**				
No risk psychological distress	96.4	6.5	26.8	7.4
High risk psychological distress	3.6	13.7	29.6	28.0
**Often of reading**				
Every day	59.5	4.7	25.5	6.2
Several times a week	18.9	6.8	28.0	8.0
Once or twice a week	14.6	10.4	30.2	11.6
Once or twice a month	26.0	12.0	29.6	16.8
Less often	18.5	14.7	31.1	17.4
Not at all	24.7	21.6	27.6	19.7
**Parenting Style**				
Firm rules and discipline	17.4	12.3	30.2	16.2
Lots of fun	1.5	8.7	31.5	7.7
Have not really thought about it	4.8	10.0	32.0	12.7
Firm discipline plus lots of fun	42.1	5.4	27.2	7.4

Doing my best for the child	34.2	7.4	26.8	8.5
**Parent's disagree child educ.**				
Never	34.7	7.8	26.7	5.9
Less than once a week	48.4	5.6	26.4	7.9
Once a week	10.1	7.5	28.9	11.8
Several times a week	5.0	7.9	30.7	16.2
At least once a day	1.7	9.5	24.6	16.2
**Regular bed time**				
Always	41.7	5.4	24.7	7.2
Usually	38.3	5.7	26.5	6.9
Sometimes	13.0	11.4	32.3	12.6
Never or almost never	7.0	11.7	32.4	12.8
**Help to learn sports**				
Yes	80.0	6.2	26.9	7.8
No	20.0	8.9	26.9	9.6
**Conflicts parents-child**				
Score 7-15, lowest conflict	43.7	6.0	26.4	3.9
Score 16-20	28.3	6.2	26.1	7.8
Score 21-26	20.1	7.8	28.0	11.7
Score 27-35, highest conflict	7.8	10.5	30.5	24.4
**Closeness parents-child**				
Score 35, highest warmth	48.3	4.7	26.6	5.5
Score 33-34	30.3	6.75	26.6	8.3
Score 30-32	15.7	9.4	28.1	11.9
Score 7-29, lowest warmth	5.7	17.0	28.1	19.7
**Hearing problems**				
No	95.3	6.7	26.9	7.9
Yes	4.7	7.4	27.5	13.6
**Concern about child's speech**				
No	87.6	6.0	26.9	7.0
Yes	12.4	11.8	27.1	16.7
**Understands child's speech**				
Always	97.7	6.4	26.9	7.8
Sometimes	1.8	18.6	26.6	22.3
Rarely	0.5	38.3	31.9	36.2
**Walk up steps**				

Yes	96.8	6.6	26.8	7.9
With help or not able	3.2	11.7	30.9	14.8
**Child disability or illness**				
No	84.6	6.5	26.9	7.3
Yes	15.4	8.3	27.1	13.1
**Naming vocabulary disability**				
No language disability	92.1	5.4	26.6	7.7
Language disability	7.9	22.9	30.2	14.1
**SDQ**				
No related problems	89.8	5.8	26.6	5.8
Socioemotional Problems	10.2	15.2	29.6	29.3
**BMI**				
Normal weight	76.5	6.7	19.5	8.0
Overweight/obesity	23.5	7.0	51.0	8.6

Additional data of Table 3- **Multivariable associations between factors included in Model 1 and 3 and language disability, overweight/obese and socioemotional behavioural problems at age 11. Millennium Cohort Study, 2001-2012, United Kingdom (imputed data, N=10,262)**

**Model 1[1]**						
Outcomes –Age 11	**Cognitive-Language disability**		**Physical- Overweight / obese**		**Behavioural- Socioemotional problems**	
Predictors	Relative Risk (95% CI)	SDS/Ranking*	Relative Risk (95% CI)	SDS/ Ranking*	Relative Risk (95% CI)	SDS/ Ranking*
**Mother ethnicity**		0.039/7		0.014/9	0.006/12	
White	Ref		Ref		Ref	
Mixed	1.08 (0.48-2.41)		0.92 (0.62-1.36)		1.73 (1.03-2.92)	
Indian	0.52 (0.28-0.95)		1.03 (0.75-1.41)		0.73 (0.38-1.39)	
Pakistani	1.51 (0.88-2.56)		1.17 (0.94-1.46)		0.84 (0.48-1.46)	
Bangladeshi	2.26 (1.28-3.99)		1.01 (0.72-1.42)		0.84 (0.41-1.72)	
Black	0.83 (0.48-1.42)		1.43 (1.19-1.73)		1.19 (0.70-2.01)	
Other	0.85 (0.44-1.61)		0.82 (0.55-1.24)		0.63 (0.25-1.57)	
**Mother's age at birth**		0.052/5		0.003/12		0.108/4
14-19 years old	1.11 (0.75-1.65)		0.88 (0.73-1.07)		1.57 (1.13-2.18)	
20-24 years old	1.28 (0.94-1.74)		0.91 (0.79-1.04)		1.70 (1.27-2.26)	
25-29 years old	1.02 (0.75-1.39)		0.93 (0.83-1.04)		1.30 (1.02-1.66)	
30-34 years old	1.05 (0.79-1.40)		0.97 (0.88-1.07)		1.06 (0.83-1.36)	
35-63 years old	Ref.		Ref		Ref.	
**Language spoke at home**		0.061/4		0.002/13		0.014/10
Only English	Ref		Ref		Ref	
English and additional language	0.82 (0.52-1.29)		1.07 (0.90-1.26)		0.89 (0.58-1.37)	
Not English	0.70 (0.40-1.24)		1.14 (0.89-1.45)		0.42 (0.22-0.78)	

**Parental employment status**		0.423/1		0.035/4		0.241/1
Both parents in work	Ref		Ref		Ref	
One parent in work	1.61 (1.13-2.30)		0.99 (0.88-1.12)		1.17 (0.90-1.52)	
Neither parent in work	2.06 (1.21-3.49)		1.06 (0.86-1.31)		1.07 (0.64-1.78)	
**Deprivation- IMD**		0.196/2		0.049/3		0.095/5
1 quintile- highest	Ref.		Ref		Ref.	
2 quintile	1.48 (0.97-2.26)		1.13 (0.96-1.32)		0.90 (0.64-1.25)	
3 quintile	1.74 (1.18-2.55)		1.13 (0.98-1.30)		1.01 (0.74- 1.38)	
4 quintile	1.68 (1.14-2.27)		1.14 (0.98-1.33)		1.45 (1.06-1.97)	
5 quintile- lowest	2.18 (1.51-3.14)		1.21 (1.04-1.41)		1.19 (0.88-1.63)	
**Child gender**		0.006/12		0.032/5		0.094/6
Male	Ref		Ref		Ref	
Female	1.11 (0.95-1.31)		1.16 (1.07-1.26)		0.63 (0.54-0.73)	
**Child birth weight**		0.002/9		0.001/15		0.005/13
Normal (>=2.5 to <=4.5 kg)	Ref		Ref		Ref	
Low (<2.5 kg)	0.99 (0.69-1.42)		0.85 (0.70-1.02)		1.32 (0.98-1.77)	
High (>4.5 kg)	1.46 (0.86-2.49)		1.26 (1.01-1.56)		0.78 (0.38-1.59)	
**Gestational age**		0.001/15		0.002/14		0.001/14
Term, 37-41 weeks	Ref		Ref		Ref	
Preterm, 23-36 weeks	1.55 (1.05-2.29)		1.08 (0.90-1.29)		1.10 (0.79-1.53)	
Post-term, 42-43 weeks	0.76 (0.57-1.01)		1.11 (1.01-1.24)		0.96 (0.78-1.19)	
**Smoking in pregnancy**		0.037/8		0.061/2		0.156/2
None	Ref		Ref		Ref	
1-10 cigarettes/day	1.02 (0.79-1.32)		1.20 (1.07-1.34)		1.30 (1.08-1.56)	
11-20 cigarettes/day	1.06 (0.70-1.62)		1.40 (1.18-1.66)		1.89 (1.46-2.45)	
> 20 cigarettes/day	1.87 (1.16-3.01)		1.33 (1.02-1.74)		1.86 (1.21-2.86)	
**Alcohol consumption in pregnancy**		0.041/6		0.029/6		0.012/11
No	Ref		Ref		Ref	
Yes	0.99 (0.79-1.23)		0.87 (0.80-0.95)		0.96 (0.81-1.13)	
**Breastfeeding initiation**		0.067/3		0.023/7		0.017/9
Yes	Ref		Ref		Ref	
No	1.09 (0.90-1.32)		1.03 (0.95-1.13)		0.97 (0.83-1.13)	
**Maternal depression or anxiety**		0.024/10		0.017/8		0.122/3
No	Ref		Ref		Ref	
Yes	1.15 (0.97-1.38)		1.08 (0.99-1.17)		1.47 (1.25-1.72)	
**Type of delivery**		0.002/13		0.006/11		0.001/15
Normal	Ref		Ref		Ref	
Assisted (forceps, vacuum, breach)	1.12 (0.77-1.63)		0.91 (0.80-1.03)		1.01 (0.73-1.39)	
Planned caesarean	1.22 (0.92-1.61)		1.06 (0.94-1.21)		0.85 (0.66-1.09)	
Emergency caesarean	1.04 (0.80-1.34)		1.03 (0.92-1.16)		1.11 (0.89-1.38)	
Other	0.90 (0.22-3.62)		0.90 (0.51-1.62)		1.09 (0.51-2.34)	
**Mother BMI before born**		0.020/11		0.707/1		0.052/8
Normal	Ref		Ref		Ref	
Overweight/ obese	1.27 (1.07-1.51)		2.00 (1.85-2.16)		1.36 (1.17-1.59)	
**Mother disability or illness**		0.001/14		0.012/10		0.072/7
No	Ref		Ref		Ref	

Yes	0.91 (0.74-1.11)		1.07 (0.98-1.17)		1.36 (1.17-1.58)	
**Hosmer-Lemeshow/ p value****	12.03/ 0.133		4.55/ 0.804		5.50/ 0.703	
**Model 3[3]**						
Outcomes –Age 11	**Cognitive-Language disability**		**Physical- Overweight / obese**		**Behavioural- Socioemotional problems**	
Predictors	Relative Risk (95% CI)	SDS/Ranking*	Relative Risk (95% CI)	SDS/Ranking*	Relative Risk (95% CI)	SDS/Ranking*
**Mother ethnicity**		0.017/11		0.004/12		0.007/20
White	Ref		Ref		Ref	
Mixed	0.97 (0.51-1.85)		0.81 (0.56-1.16)		1.76 (0.95-3.26)	
Indian	0.29 (0.14-0.57)		1.20 (0.88-1.64)		0.88 (0.44-1.76)	
Pakistan	0.77 (0.53-1.12)		1.39 (1.18-1.63)		0.85 (0.50-1.43)	
Bangladeshi	0.81 (0.50-1.31)		1.08 (0.80-1.46)		0.83 (0.42-1.62)	
Black	0.64 (0.37-1.11)		1.32 (1.10-1.58)		1.09 (0.66-1.80)	
Other	0.43 0.19-0.96)		0.95 (0.69-1.30)		0.43 (0.17-1.07)	
**Mother's age at birth**		0.028/10		-		0.019/13
14-19 years old	1.30 (0.84-2.00)		-		1.15 (0.82-1.61)	
20-24 years old	1.38 (1.01-1.87)		-		1.34 (1.00-1.80)	
25-29 years old	1.11 (0.83-1.49)		-		1.18 (0.92-1.51)	
30-34 years old	1.11 (0.83-1.49)		-		1.02 (0.80-1.30)	
35-63 years old	Ref		-		Ref	
**Maternal education**		0.230/1		0.029/3		0.027/9
Degree plus	Ref		Ref		Ref	
Diploma	2.55 (1.46-4.45)		1.22 (1.06-1.42)		1.66 (1.12-2.46)	
A-levels	2.53 (1.45-4.01)		1.19 (1.02-1.38)		1.79 (1.25-2.57)	
GCSE Grades A-C	3.74 (2.48-5.63)		1.34 (1.20-1.54)		1.71 (1.18-2.46)	
GCSE Grades D-G	4.49 (2.85-7.07)		1.26 (1.08-1.48)		1.72 (1.18-2.51)	
None of these qualifications	4.67 (2.95-7.39)		1.31 (1.11-1.54)		1.61 (1.07-2.42)	
**Parents employment status**		-		0.010/10		0.032/7
Both parents in work	-		Ref		Ref	
One parent in work	-		0.98 (0.82-1.18)		1.02 (0.70-1.23)	
Neither parent in work	-		0.97 (0.61-1.54)		2.42 (1.15-4.86)	
**Deprivation- IMD**		0.062/6		0.017/5		0.011/18
1 quintile- highest	Ref		Ref		Ref	
2 quintile	1.39 (0.90-2.13)		1.13 (0.95-1.31)		0.83 (0.59-1.16)	
3 quintile	1.30 (0.87-1.95)		1.09 (0.96-1.27)		0.87 (0.64-1.20)	
4 quintile	1.24 (0.83-1.84)		1.09 (0.97-1.26)		1.20 (0.89-1.61)	
5 quintile- lowest	1.26 (0.86-1.85)		1.15 (0.99-1.36)		0.89 (0.65-1.22)	
**Child gender**		0.007/14		0.014/7		
Male	Ref		Ref		Ref	0.027/10
Female	1.19 (1.00-1.40)		1.15 (1.06-1.25)		0.67 (0.58-0.79)	
**Number of siblings**		0.044/8				-
1-2	Ref		-		-	
3 or more	1.32 (1.10-1.59)		-		-	
**Income**		0.115/3		-		0.027/8
Above 60%	Ref		-		Ref	
Below 60%, poverty	1.42 (1.14-1.76)		-		1.21 (1.00-1.45)	

**Language spoke at home**		-		-		
Only English	-		-		Ref	0.012/16
English and additional language	-		-		0.66 (0.44-0.99)	
Not English					0.31 (0.15-0.61)	
**Gestational age**		0.001/15		-		-
Term, 37-41 weeks	Ref		-		-	
Preterm, 23-36 weeks	1.41 (1.06-1.86)		-		-	
Post-term, 42-43 weeks	0.84 (0.63-1.11)		-		-	
**Child birth weight**		-		-		0.001/22
Normal (>=2.5 to <=4.5 kg)	-		-		Ref	
Low (<2.5 kg)	-		-		1.31 (1.06-1.62)	
High (>4.5 kg)	-		-		0.75 (0.36-1.57)	
**Smoking in pregnancy**		-		0.023/4		0.039/6
None	-		Ref		Ref	
1-10 cigarettes/day	-		1.14 (1.02-1.27)		1.09 (0.90-1.31)	
11-20 cigarettes/day	-		1.32 (1.13-1.54)		1.35 (1.03-1.76)	
> 20 cigarettes/day	-		1.40 (1.05-1.86)		1.13 (0.71-1.78)	
**Alcohol consumption in pregnancy**		-		0.011/9		-
No	-		Ref		-	
Yes	-		0.89 (0.83-0.96)		-	
**Maternal general health**		-				0.040/5
Excellent	-		Ref	0.015/6	Ref	
Good	-		0.97 (0.89-1.06)		1.34 (1.10-1.63)	
Fair	-		1.13 (1.00-1.28)		1.29 (1.02-1.63)	
Poor	-		1.09 (0.89-1.33)		1.66 (1.17-2.34)	
**Maternal depression or anxiety**		-		-		0.024/16
No	-		-		Ref	
Yes	-		-		1.20 (1.02-1.42)	
**Maternal mental health at age 3**		-		-		0.083/3
No risk psychological distress	-		-		Ref	
High risk psychological distress	-		-		1.40 (1.09-1.80)	
**Mother BMI before born**		-		0.291/2		0.015/14
Normal	-		Ref		Ref	
Overweight/ obese	-		1.83 (1.69-2.01)		1.30 (1.13-1.50)	
**Mother disability or illness**		-		0.005/11		0.014/15
No	-		Ref		Ref	
Yes	-		1.05 (0.96-1.15)		1.15 (0.97-1.35)	
**Parental Style**		-		0.002/13		0.002/21
Firm rules and discipline	-		0.98 (0.76-1.26)		1.35 (0.91-2.01)	
Lots of fun	-		1.17 (1.00-1.35)		0.76 (0.52-1.11)	
Have not really thought about it	-		1.02 (0.80-1.30)		0.98 (0.59-1.60)	
Firm discipline plus lots of fun	-		Ref		Ref	
Doing my best for the child	-		0.97 (0.90-1.05)		0.87 (0.74-1.03)	
**Lone parenthood**		-		-		0.024/11
No	-		-		Ref	
Yes	-		-		1.15 (0.96-1.38)	

**Parents disagree about child**		0.007/13		-		-
Never	Ref		-		-	
Less than once a week	0.82 (0.67-1.01)		-		-	
Once a week	0.79 (0.54-1.16)		-		-	
Several times a week	0.63 (0.35-1.13)		-		-	
At least once a day	0.99 (0.52-1.88)		-		-	
**Regular bed time**		0.039/9		0.013/8		0.008/19
Always	Ref		Ref		Ref	
Usually	1.17 (0.95-1.44)		1.10 (1.01-1.20)		0.94 (0.78-1.13)	
Sometimes	1.25 (0.99-1.56)		1.23 (1.09-1.39)		1.24 (1.02-1.50)	
Never or almost never	1.59 (1.19-2.11)		1.14 (0.98-1.32)		0.82 (0.63-1.07)	
**Conflicts parents-child age 3**		-		-		0.161/2
Score 7-15, lowest conflict	-		-		Ref	
Score 16-20	-		-		1.49 (1.17-1.88)	
Score 21-26	-		-		1.72 (1.34-2.21)	
Score 27-35, highest conflict	-		-		2.14 (1.63-2.82)	
**Closeness parents-child age 3**		0.087/4		-		-
Score 35, highest warmth	Ref		-		-	
Score 33-34	1.30 (1.00-1.70)		-		-	
Score 30-32	1.30 (0.97-1.76)		-		-	
Score 7-29, lowest warmth	1.76 (1.21-2.57)		-		-	
**Often of reading age 3**		0.074/5		0.002/14		0.041/4
Every day	Ref		Ref		Ref	
Several times a week,	1.19 (0.93-1.53)		1.00 (0.91-1.09)		1.08 (0.85-1.36)	
Once or twice a week,	1.15 (0.91-1.46)		0.96 (0.87-1.07)		1.21 (1.01-1.45)	
Once or twice a month,	1.33 (0.86-2.07)		1.01 (0.79-1.28)		1.54 (1.06-2.24)	
Less often	1.44 (0.95-2.18)		0.89 (0.68-1.15)		1.21 (0.87-1.68)	
Not at all	1.24 (0.81-1.90)		0.75 (0.57-0.98)		1.64 (1.21-2.23)	
**Naming vocabulary disability age 3**		0.214/2		-		0.012/17
No language disability	Ref		-		Ref	
Language disability	2.27 (1.77-2.92)		-		1.16 (0.90-1.50)	
**SDQ age 3**		0.060/7		-		0.364/1
No related problems	Ref		-		Ref	
Behavioural problems	1.29 (1.01-1.64)		-		2.09 (1.69-2.59)	
**BMI age 3**		-		0.557/1		-
Normal weight	-		Ref		-	
Overweight/obese	-		2.46 (2.27-2.67)		-	
**Hosmer-Lemeshow/ p value****	12.03/ 0.150		7.88/ 0.445		14.47/ 0.070	

[1]Model 1 includes information collected in maternity services in England; [3] Model 3 shown final parsimonious models that includes variables statistically selected from the initial saturated model (30 variables): 14 variables for language disability, 14 for overweight/ obese and 22 for socioemotional behavioural problems.
*Standardised Dominance Statistic (SDS) and weighted ranking of predictive risk variables; **Calibration analyses.

Prevalence of early childhood predictors and language disability, overweight /obesity and socioemotional behavioural problems outcomes at age 11. Response sample, complete cases and imputed data.

	Response sample		Complete cases (3,798)	Imputed (10,262)
	(n)	(%)	(%)	(%)
**Outcomes -Age 11**				
Cognitive- language disability	12,824	7.8	4.8	6.7
Physical- overweight/obesity	12,654	26.9	24.1	26.9
Behavioural- socioemotional problems	12,584	10.4	6.5	8.2
**Perinatal predictors**				
Child gender, male	15,561	52.6	50.0	50.4
Mother ethnicity, not white[1]	13,537	12.5	7.0	11.9
Number siblings, ≥ 3	15,561	23.0	19.7	22.4
Language spoke at home, not English[2]	15,561	11.9	5.4	11.3
Mother age at birth, <20 years	15,538	11.8	3.9	6.7
Maternal education, lowest qualification	15,637	23.0	8.2	14.8
Neither parents in work	12,895	9.6	3.7	7.6
Lone parenthood	16,172	19.2	3.2	13.5
Income, <60% poverty	15,508	39.7	16.7	30.1
Deprivation IMD, lowest quintile	8,500	27.3	15.0	25.6
Birth weight, ≠ normal[3]	13,531	8.1	7.0	7.9
Gestational age, preterm	13,428	6.0	5.4	5.7
Initiated breastfeeding	15,525	38.3	24.2	30.0
Smoked in pregnancy[4]	15,481	26.9	16.5	20.8
Consumed alcohol in pregnancy	13,541	30.7	37.2	31.9
Mothers had disability or illness	15,539	21.5	21.1	22.0
Type of delivery, not normal[5]	15,525	28.8	30.5	31.8
Mother general health, poor	15,541	3.1	2.0	2.6
Mother had depression or anxiety	15,541	25.8	22.1	24.4
Mother BMI before born, overweight/obesity	14,196	27,7	28,6	29.4
**Predictors- Age 3**				
Mother had mental health disorders	12,591	3.6	2.3	3.6
Parents-child closeness, lowest warmth	11,827	6.2	3.8	5.7
Parents-child conflict, highest conflict	12,077	8,6	7.1	7.8
Parenting style, only lots of fun	13,548	2.0	1.3	1.5

Parents had any disagreement about child[6]	10,383	64.2	63.3	65.3
No help to child learn sports	14,107	21.3	18.0	20.0
Often of reading, not every day[7]	14,107	44.1	34.8	40.5
Regular bed time, not always[8]	14,107	59.4	55.5	58.3
Child had hearing problems	14,024	4.6	5.2	4.7
Had concerns about child speech	14,107	14.3	10.8	12.4
Can understand child speak, sometimes/ never	14,107	3.1	1.4	2.2
Child cannot walk up steps alone[9]	14,089	3.3	2.7	3.1
Child had disability or illness	14,106	16.1	16.1	15.4
Child had language disability	13,312	8.7	3.8	7.9
Child had socioemotional problems	13,177	12.4	7.6	10.2
Child overweight/obesity	12,079	23.4	21.4	23.5

[1] not white: mixed, Indian, Pakistani, Bangladeshi, Black and other; [2] not English: not only English or other languages; [3]≠ normal: low or over birth weight; [4] smoked in pregnancy: 1-10/ 11-20 and >20 cigarettes/day; [5] not normal: assisted (forceps, vacuum, breach), planned caesarean, emergency caesarean and other; 6 Any disagreement about child: less than once a week, once a week, several times a week and at least once a day; [7]not every day: several times a week, once or twice a week, once or twice a month, less often and not at all; [8] not always: usually, sometimes, never almost never; [9] Child cannot walk up steps alone: with help or not able.

Complete case analyses

Multivariable associations between factors included in Model 1, 2 and 3 and language disability, overweight/obese and socioemotional behavioural problems at age 11 (complete cases, N=3,798)

**Model 1[1]**						
Outcomes –Age 11	**Cognitive-Language disability**		**Physical- Overweight / obese**		**Behavioural- Socioemotional problems**	
Predictors	Relative Risk (95% CI)	Ranking*	Relative Risk (95% CI)	Ranking*	Relative Risk (95% CI)	Ranking*
**Mother ethnicity**		12		6		14
White	Ref		Ref		Ref	
Mixed	0.68 (0.87-5.34)		0.51 (1.05-1.57)		2.95 (1.02-8.54)	
Indian	0.17 (0.02-1.35)		1.10 (0.67-1.80)		0.48 (0.08-2.68)	
Pakistani	1.46 (0.47-4.45)		1.15 (0.78-1.68)		0.52 (0.15-1.73)	
Bangladeshi	1.98 (0.32-12.18)		1.09 (0.44-2.69)		2.20 (0.53-9.03)	
Black	0.61 (0.14-2.63)		1.49 (1.07-2.09)		1.36 (0.55-3.35)	
Other	1.08 (0.23-5.06)		0.89 (0.41-1.93)		0.22 (0.03-1.49)	
**Mother's age at birth**		7		11		2
14-19 years old	1.12 (0.42-2.95)		0.60 (0.37-0.97)		1.72 (0.85-3.46)	
20-24 years old	1.25 (0.69-2.26)		0.83 (0.66-1.05)		2.27 (1.37-3.75)	
25-29 years old	1.21 (0.74-1.99)		0.92 (0.78-1.09)		1.86 (1.20-2.86)	
30-34 years old	1.15 (0.71-1.87)		0.96 (0.84-1.11)		1.01 (0.63-1.61)	
35-63 years old	Ref.		Ref		Ref.	
**Language spoke at home**		14		15		13

Only English	Ref		Ref		Ref	
English and additional language	1.03 (0.39-2.70)		1.13 (0.82-1.55)		1.08 (0.39-2.95)	
Not English	0.37 (0.40-3.59)		0.78 (0.30-2.02)		1.98 (0.53-7.38)	
**Parents employment status**		1		5		3
Both parents in work	Ref		Ref		Ref	
One parent in work	1.29 (0.93-1.81)		1.02 (0.90-1.15)		0.92 (0.66-1.28)	
Neither parent in work	1.97 (1.12-3.47)		1.07 (0.81-1.42)		1.69 (1.11-2.58)	
**Deprivation- IMD**		2		2		5
1 quintile- highest	Ref.		Ref		Ref.	
2 quintile	1.30 (0.71-2.38)		1.14 (0.90-1.44)		0.90 (0.53-1.53)	
3 quintile	1.51 (0.84-2.69)		1.22 (1.01-1.48)		0.89 (0.54- 1.46)	
4 quintile	1.59 (0.89-2.84)		1.09 (0.89-1.35)		1.47 (0.93-2.32)	
5 quintile- lowest	2.26 (1.26-4.07)		1.32 (1.08-1.62)		1.44 (0.89-2.31)	
**Child gender**		10		4		8
Male	Ref		Ref		Ref	
Female	1.13 (0.86-1.48)		1.18 (1.03-1.35)		0.68 (0.53-0.88)	
**Child birth weight**		8		10		15
Normal (>=2.5 to <=4.5 kg)	Ref		Ref		Ref	
Low (<2.5 kg)	1.17 (0.63-2.19)		0.86 (0.59-1.25)		1.05 (0.54-2.02)	
High (>4.5 kg)	1.04 (0.31-3.41)		1.56 (1.13-2.14)		0.38 (0.07-1.94)	
**Gestational age**		13		14		12
Term, 37-41 weeks	Ref		Ref		Ref	
Preterm, 23-36 weeks	1.73 (0.99-3.04)		1.25 (0.91-1.74)		1.26 (0.67-2.35)	
Post-term, 42-43 weeks	0.87 (0.48-1.57)		1.10 (0.92-1.32)		0.84 (0.55-1.31)	
**Smoking in pregnancy**		3		7		1
None	Ref		Ref		Ref	
1-10 cigarettes/day	0.92 (0.59-1.46)		1.16 (0.95-1.40)		1.28 (0.90-1.82)	
11-20 cigarettes/day	2.08 (1.16-3.72)		1.12 (0.80-1.56)		2.77 (1.72-4.47)	
> 20 cigarettes/day	1.01 (0.18-5.53)		1.63 (0.94-2.83)		2.98 (0.95-9.36)	
**Alcohol consumption in pregnancy**		4		3		9
No	Ref		Ref		Ref	
Yes	0.74 (0.51-1.06)	9	0.88 (0.77-1.01)		0.84 (0.62-1.15)	
**Breastfeeding initiation**				9		10
Yes	Ref		Ref		Ref	
No	0.97 (0.69-1.37)		1.11 (0.98-1.26)		0.97 (0.72-1.44)	
**Maternal depression or anxiety**		5		13		4
No	Ref		Ref		Ref	
Yes	1.24 (0.85-1.80)		1.04 (0.90-1.20)		1.24 (0.98-1.31)	
**Type of delivery**		11		12		11
Normal	Ref		Ref		Ref	
Assisted (forceps, vacuum, breach)	1.00 (0.55-1.82)		0.82 (0.67-1.00)		1.00 (0.66-1.51)	
Planned caesarean	1.34 (0.85-2.12)		1.07 (0.89-1.29)		1.09 (0.71-1.69)	
Emergency caesarean	0.83 (0.51-1.36)		1.00 (0.82-1.22)		1.02 (0.67-1.56)	
Other	2.31 (0.65-8.20)		1.48 (0.81-2.69)		1.45 (0.45-4.66)	
**Mother BMI before born**		6		1		7
Normal	Ref		Ref		Ref	

Overweight/ obese	1.25 (0.89-1.75)		2.16 (1.91-2.45)		1.47 (1.12-1.94)	
**Mother disability or illness**		15		8		6
No	Ref		Ref		Ref	
Yes	0.96 (0.65-1.342)		1.11 (0.82-1.55)		1.60 (1.22-2.09)	
**Hosmer-Lemeshow/ p value****	7.31/ 0.503		6.30/ 0.614		2.21/ 0.974	
**Model 2[2]**						
Outcomes –Age 11	**Cognitive-Language disability**		**Physical- Overweight / obese**		**Behavioural- Socioemotional problems**	
Predictors	Relative Risk (95% CI)	Ranking*	Relative Risk (95% CI)	Ranking*	Relative Risk (95% CI)	Ranking*
**Mother ethnicity**		18		7		20
White	Ref		Ref		Ref	
Mixed	0.46 (0.06-3.50)		0.59 (0.25-1.41)		2.71 (0.96-7.59)	
Indian	0.18 (0.02-1.32)		1.19 (0.74-1.90)		0.51 (0.10-2.41)	
Pakistani	0.67 (0.26-1.71)		1.22 (0.82-1.80)		0.40 (0.12-1.33)	
Bangladeshi	1.12 (0.31-4.06)		1.09 (0.47-2.50)		2.14 (0.51-8.96)	
Black	0.32 (0.07-1.49)		1.37 (1.07-1.76)		1.34 (0.52-3.42)	
Other	0.34 (0.08-1.51)		0.94 (0.43-2.02)		0.21 (0.03-1.46)	
**Mother's age at birth**		12		12		4
14-19 years old	1.41 (0.72-2.77)		0.59 (0.37-0.96)		1.48 (0.74-2.95)	
20-24 years old	0.84 (0.48-1.47)		0.88 (0.71-1.10)		2.09 (1.26-3.46)	
25-29 years old	0.99 (0.60-1.63)		0.91 (0.77-1.07)		1.82 (1.17-2.85)	
30-34 years old	1.09 (0.68-1.75)		0.99 (0.86-1.14)		1.04 (0.63-1.69)	
35-63 years old	Ref		Ref		Ref	
**Language spoke at home**		16		19		16
Only English	Ref		Ref		Ref	
English and additional language	0.99 (0.49-1.99)		1.06 (0.79-1.42)		1.16 (0.44-3.02)	
Not English	0.19 (0.02-1.47)		0.96 (0.40-2.34)		1.58 (0.42-5.96)	
**Parents employment status**		3		6		5
Both parents in work	Ref		Ref		Ref	
One parent in work	1.25 (0.88-1.77)		1.07 (0.94-1.20)		0.89 (0.63-1.25)	
Neither parent in work	1.81 (0.99-3.30)		1.23 (0.93-1.63)		1.52 (0.95-2.42)	
**Deprivation- IMD**		4		5		9
1 quintile- highest	Ref		Ref		Ref	
2 quintile	1.41 (0.83-2.40)		1.11 (0.88-1.40)		0.92 (0.54-1.58)	
3 quintile	1.30 (0.79-2.13)		1.18 (0.97-1.43)		0.88 (0.53-1.47)	
4 quintile	1.37 (0.83-2.28)		1.06 (0.86-1.31)		1.46 (0.93-2.27)	
5 quintile- lowest	2.10 (1.18-3.42)		1.24 (1.08-1.62)		1.34 (0.82-2.20)	
**Child gender**		15		3		12
Male	Ref		Ref		Ref	
Female	1.24 (0.95-1.62)		1.17 (1.02-1.34)		0.84 (0.65-1.08)	
**Child birth weight**		14		10		21
Normal (>=2.5 to <=4.5 kg)	Ref		Ref		Ref	
Low (<2.5 kg)	0.96 (0.48-1.93)		0.99 (0.71-1.39)		0.95 (0.49-1.84)	
High (>4.5 kg)	1.11 (0.32-3.78)		1.33 (0.93-1.88)		0.37 (0.06-2.11)	
**Gestational age**		21		17		23
Term, 37-41 weeks	Ref		Ref		Ref	

Preterm, 23-36 weeks	1.68 (0.92-3.08)		1.20 (0.88-1.65)		1.05 (0.52-2.12)	
Post-term, 42-43 weeks	0.85 (0.47-1.53)		1.09 (0.93-1.29)		0.91 (0.60-1.37)	
**Smoking in pregnancy**		5		9		2
None	Ref		Ref		Ref	
1-10 cigarettes/day	0.89 (0.56-1.39)		1.13 (0.94-1.34)		1.14 (0.81-1.61)	
11-20 cigarettes/day	1.58 (0.82-3.03)		1.20 (0.87-1.64)		2.13 (1.29-3.51)	
> 20 cigarettes/day	1.06 (0.17-6.33)		1.86 (1.01-3.42)		2.47 (0.80-7.61)	
**Alcohol consumption in pregnancy**		6		4		15
No	Ref		Ref		Ref	
Yes	0.76 (0.53-1.08)		0.88 (0.78-0.99)		0.87 (0.63-1.19)	
**Breastfeeding initiation**		13		11		18
Yes	Ref		Ref		Ref	
No	0.95 (0.69-1.32)		1.03 (0.90-1.20)		0.99 (0.74-1.32)	
**Maternal depression or anxiety**		9		14		6
No	Ref		Ref		Ref	
Yes	1.27 (0.93-1.72)		1.04 (0.90-1.19)		1.33 (0.98-1.81)	
**Type of delivery**		27		13		19
Normal	Ref		Ref		Ref	
Assisted (forceps, vacuum, breach)	1.41 (0.72-2.77)		0.80 (0.65-0.99)		1.08 (0.71-1.65)	
Planned caesarean	1.30 (0.82-2.07)		1.07 (0.88-1.29)		0.92 (0.59-1.44)	
Emergency caesarean	0.83 (0.50-1.39)		0.97 (0.80-1.17)		1.05 (0.68-1.64)	
Other	2.68 (0.71-10.05)		1.21 (0.66-2.22)		1.66 (0.49-5.61)	
**Mother BMI before born**		8		2		10
Normal	Ref		Ref		Ref	
Overweight/ obese	1.20 (0.88-1.64)		1.95 (1.73-2.20)		1.36 (1.03-1.79)	
**Mother disability or illness**		23		8		7
No	Ref		Ref		Ref	
Yes	0.88 (0.61-1.27)		1.16 (1.02-1.31)		1.48 (1.12-1.95)	
**Hearing problems age 3**		19		20		22
No	Ref		Ref		Ref	
Yes	1.26 (0.60-2.63)		1.22 (0.96-1.56)		0.78 (0.47-1.29)	
**Concern about child's speech age 3**		10		16		3
No	Ref		Ref		Ref	
Yes	0.83 (0.48-1.45)		0.91 (0.74-1.13)		1.93 (1.43-2.61)	
**Understands child's speech age 3**		7		21		14
Always	Ref		Ref		Ref	
Sometimes	2.31 (0.64-8.30)		0.78 (0.47-1.29)		0.97 (0.39-2.41)	
Rarely	6.17 (0.62-61.16)		1.84 (0.66-5.09)		6.06 (2.22-7.89)	
**Walk up steps age 3**		20		22		13
Yes	Ref		Ref		Ref	
With help	1.27 (0.48-3.38)		0.53 (0.28-0.99)		1.48 (0.78-2.80)	
No	0.48 (0.10-2.20)		1.18 (0.75-1.85)		1.27 (0.45-3.58)	
**Child disability or illness age 3**		11		23		8
No	Ref		Ref		Ref	
Yes	1.25 (0.85-1.84)		1.01 (0.88-1.16)		1.50 (1.09-2.05)	
**Naming vocabulary disability age 3**		1		15		11

No language disability	Ref		Ref		Ref	
Language disability	1.89 (1.03-3.45)		0.97 (0.78-1.21)		1.13 (0.66-1.94)	
**SDQ age 3**		2		18		1
No related problems	Ref		Ref		Ref	
Behavioural problems	1.96 (1.32-2.93)		1.11 (0.89-1.39)		3.28 (2.28-4.72)	
**BMI age 3**		22		1		17
Normal weight	Ref		Ref		Ref	
Overweight/obese	0.87 (0.56-1.34)		2.55 (2.24-2.91)		1.23 (0.93-1.63)	
**Hosmer-Lemeshow/ p value****	13.85/ 0.086		12.00/ 0.151		5.76/ 0.674	
**Model 3[3]**						
Outcomes –Age 11	**Cognitive-Language disability**		**Physical- Overweight / obese**		**Behavioural- Socioemotional problems**	
Predictors	Relative Risk (95% CI)	Ranking*	Relative Risk (95% CI)	Ranking*	Relative Risk (95% CI)	Ranking*
**Mother ethnicity**		12		9		15
White	Ref		Ref		Ref	
Mixed	0.43 (0.06-2.88)		0.59 (0.25-1.41)		3.64 (1.19-11.13)	
Indian	0.22 (0.03-1.54)		1.17 (0.72-1.90)		0.56 (0.16-1.88)	
					0.43 (0.17-1.11)	
					2.35 (0.71-7.80)	
Black	0.30 (0.06-1.36)		1.37 (1.05-1.79)		1.60 (0.64-4.00)	
Other	0.54 0.13-2.17)		0.83 (0.39-1.76)	-	0.24 (0.03-1.62)	
**Mother's age at birth**		-				7
14-19 years old	-		-		1.17 (0.52-2.62)	
20-24 years old	-		-		1.84 (1.08-3.13)	
25-29 years old	-		-		1.84 (1.16-2.92)	
30-34 years old	-		-		0.95 (0.59-1.54)	
35-63 years old	-		-		Ref	
**Maternal education**		1		3		-
Degree plus	Ref		Ref		-	
Diploma	2.35 (1.01-5.43)		1.33 (1.09-1.64)		-	
A-levels	1.47 (0.54-4.00)		1.23 (0.97-1.56)		-	
GCSE Grades A-C	2.94 (1.49-5.77)		1.42 (1.19-1.70)		-	
GCSE Grades D-G	6.34 (2.94-13.69)		1.35 (1.10-1.65)		-	
None of these qualifications	3.96 (1.74-8.99)		1.53 (1.19-1.96)		-	
**Parents employment status**		2		-		6
Both parents in work	Ref		-		Ref	
One parent in work	1.01 (0.70-1.47)		-		0.98 (0.70-1.37)	
Neither parent in work	1.27 (0.62-2.60)		-		1.53 (0.82-2.85)	
**Deprivation- IMD**		-		7		9
1 quintile- highest	-		Ref		Ref	
2 quintile	-		1.13 (0.89-1.43)		0.97 (0.57-1.67)	
3 quintile	-		1.16 (0.95-1.41)		0.86 (0.53-1.39)	
4 quintile	-		1.04 (0.83-1.31)		1.39 (0.89-2.17)	
5 quintile- lowest	-		1.21 (0.97-1.51)		1.22 (0.74-2.00)	

**Child gender**		-		6		12
Male	-		Ref		Ref	
Female	-		1.18 (1.03-1.35)		0.78 (0.60-1.00)	
**Number of siblings**		7		-		13
1-2	Ref		-		Ref	
3 or more	1.66 (1.16-2.38)		-		1.38 (0.99-1.92)	
**Income**		3		-		
Above 60%	Ref		-		-	
Below 60%, poverty	1.59 (1.13-2.24)		-		-	
**Gestational age**		13		-		-
Term, 37-41 weeks	Ref		-		-	
Preterm, 23-36 weeks	1.62 (1.03-2.54)		-		-	
Post-term, 42-43 weeks	0.86 (0.48-1.55)		-		-	
**Smoking in pregnancy**		9		-		3
None	Ref		-		Ref	
1-10 cigarettes/day	0.68 (0.45-1.04)		-		1.17 (0.85-1.62)	
11-20 cigarettes/day	1.37 (0.74-2.51)		-		1.74 (1.01-3.00)	
> 20 cigarettes/day	0.83 (0.12-5.73)		-		1.92 (0.58-6.36)	
**Alcohol consumption in pregnancy**		-		5		-
No	-		Ref		-	
Yes	-		0.90 (0.79-1.02)		-	
**Maternal general health**		11		-		5
Excellent	Ref		-		Ref	
Good	1.27 (0.88-1.83)		-		1.17 (0.83-1.64)	
Fair	0.95 (0.58-1.55)		-		1.12 (0.69-1.81)	
Poor	0.97 (0.34-2.73)		-		1.74 (0.97-3.12)	
**Maternal mental health at age 3**		-		-		4
No risk psychological distress	-		-		Ref	
High risk psychological distress	-		-		2.08 (1.32-3.28)	
**Mother BMI before born**		-		2		11
Normal	-		Ref		Ref	
Overweight/ obese	-		2.00 (1.77-2.25)		1.41 (1.08-1.83)	
**Mother disability or illness**		-		8		10
No	-		Ref		Ref	
Yes	-		1.19 (1.05-1.36)		1.35 (1.01-1.82)	
**Parental Style**		-		10		-
Firm rules and discipline	-		0.84 (0.53-1.33)		-	
Lots of fun	-		1.27 (1.00-1.61)		-	
Have not really thought about it	-		1.07 (0.71-1.60)		-	
Firm discipline plus lots of fun	-		Ref		-	
Doing my best for the child			0.93 (0.83-1.05)			
**Lone parenthood**		-		-		8
No	-		-		Ref	
Yes	-		-		1.73 (1.08-2.79)	
**Parents disagree about child**		-		11		-
Never			Ref		-	

Less than once a week	-		0.92 (0.83-1.03)		-	
Once a week	-		0.89 (0.72-1.10)		-	
Several times a week	-		0.98 (0.73-1.31)		-	
At least once a day	-		0.44 (0.22-0.90)		-	
**Regular bed time**		6		4		14
Always	Ref		Ref		Ref	
Usually	1.30 (0.89-1.89)		1.09 (0.96-1.25)		0.74 (0.55-1.00)	
Sometimes	1.48 (0.97-2.27)		1.31 (1.10-1.56)		1.19 (0.82-1.74)	
Never or almost never	2.45 (1.40-4.28)		1.15 (0.91-1.45)		0.54 (0.31-0.96)	
**Help to learn sports**		10		-		-
Yes	Ref		-		-	
No	1.45 (1.05-2.02)		-		-	
**Conflicts parents-child age 3**		-		-		2
Score 7-15, lowest conflict	-		-		Ref	
Score 16-20	-		-		1.80 (1.26-2.58)	
Score 21-26	-		-		1.99 (1.28-3.09)	
Score 27-35, highest conflict	-		-		2.75 (1.72-4.39)	
**Closeness parents-child age 3**		5		-		-
Score 35, highest warmth	Ref		-		-	
Score 33-34	1.76 (1.26-2.47)		-		-	
Score 30-32	1.32 (0.86-2.03)		-		-	
Score 7-29, lowest warmth	2.19 (1.31-3.67)		-		-	
**Naming vocabulary disability age 3**		4		-		-
No language disability	Ref		-		-	
Language disability	1.85 (1.05-3.26)		-		-	
**SDQ age 3**		8		-		1
No related problems	Ref		-		Ref	
Behavioural problems	1.41 (0.88-2.24)		-		2.69 (1.81-3.98)	
**BMI age 3**		-		1		-
Normal weight	-		Ref		-	
Overweight/obese	-		2.53 (2.22-2.89)		-	
**Hosmer-Lemeshow/ p value****	9.41/ 0.308		12.34/ 0.136		10.53/ 0.229	

[1]Model 1 includes information collected in maternity services in England; [2]Model 2 includes information collected in maternity services in England plus correspondent factors assessed in MCS at age 3 that are collected on 2.5 year old health check in England; [3]Model 3 shown final parsimonious models that includes variables statistically selected from the initial saturated model (30 variables): 13 variables for language disability, 11 for overweight/ obese and 15 for socioemotional behavioural problems.
*dominance analysis showing weighting of each predictor variable; **calibration analyses.

AUC for three predictive models of language disability, overweight/obesity and socioemotional behavioural problems at age 11 for UK children (complete cases, N=3,798)

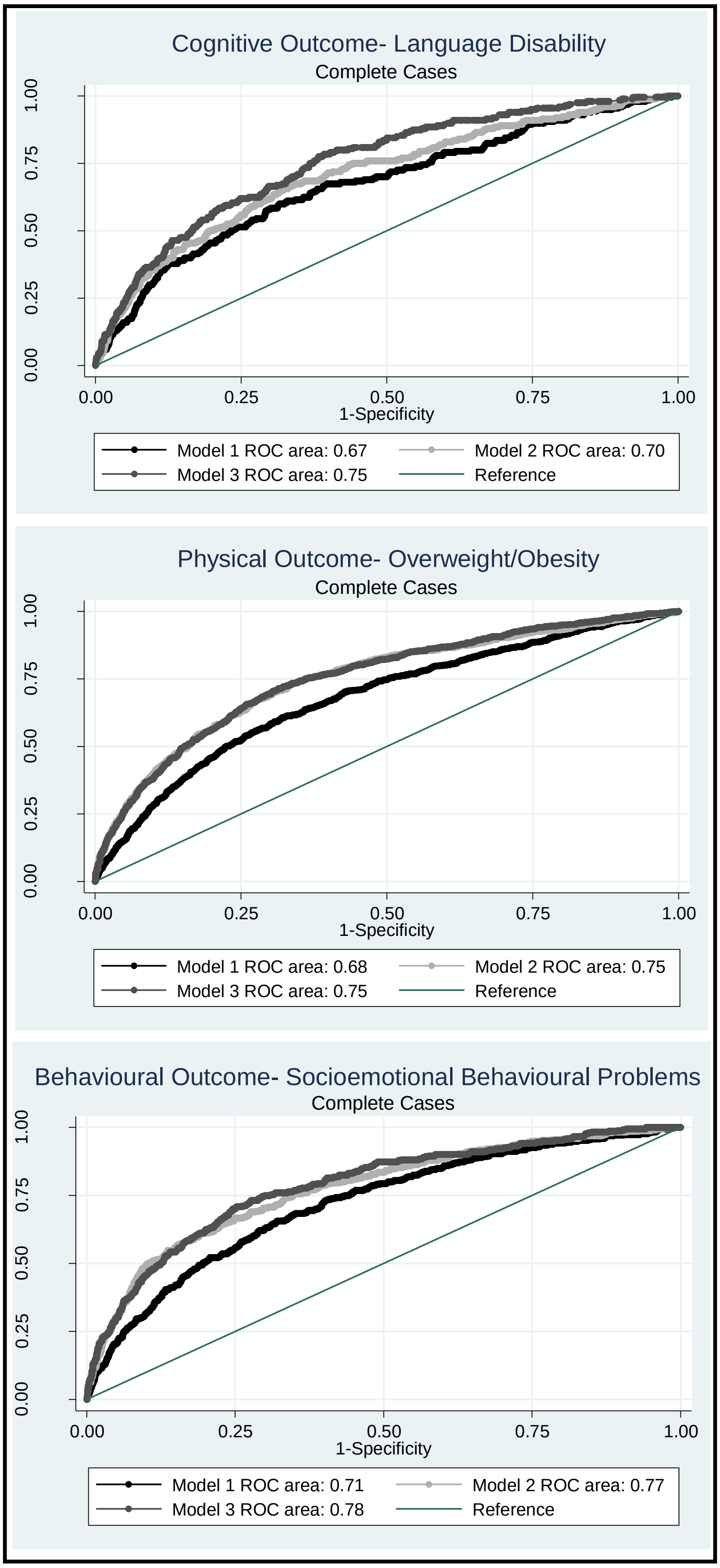

Test properties of maximised cut off probability for language disability, overweight/obese and socioemotional behavioural problems at age 11 (complete cases, N=3,798)

Test properties	Maximised cut offs								
	Cognitive<br>Language disability (%)[1]			Physical<br>Overweight/obese (%)[2]			Behavioural<br>Socioemotional problems (%)[3]		
	Model 1	Model 2	Model 3	Model 1	Model 2	Model 3	Model 1	Model 2	Model 3
Sensitivity	37.4	43.6	54.8	59.6	68.2	69.4	49.6	58.1	62.4
Specificity	85.9	87.9	83.3	69.1	71.0	70.3	83.0	87.1	84.6
PPV	11.6	15.1	14.0	38.2	42.9	42.9	16.0	22.9	21.0
NPV	96.5	96.9	97.4	84.2	87.4	87.7	96.2	96.9	97.2
% of positives	10.1	10.2	18.5	37.8	38.5	39.3	19.0	15.7	18.3
Correctly classified	83.6	85.8	82.0	66.8	70.3	70.1	80.9	85.3	83.2

PPV: positive predictive value; NPV: negative predictive value; correctly classified: true positives plus true negatives; % of positives: total of children classified as positive, even if it is true or not. [1]maximased cut offs used for language disability (model 1: 0.08, model 2: 0.08, model 3: 0.07); [2]maximased cut offs used for overweight/obese (model 1: 0.23, model 2: 0.24, model 3: 0.24); [3]maximased cut offs used for socioemotional behavioural problems (model 1: 0.09, model 2: 0.09, model 3: 0.08)

Analyses without prior outcome at age 3 years (complete case sample)

We compared the discriminatory capacity of the three models without the same predictors at age 3 of the outcomes at age 11. The models 2 and 3, excluding the outcomes at age 3, remain with predictive power about 70% and 75%, respectively, for language disability outcome, comparing when these were included. Similar patterns were observed in models 2 and 3 for socioemotional behavioural problems. However for overweight/ obese outcomes, the predictive power for this outcome would was lower without the age 3 measure of the outcome (about 68% for models 2 and 3) comparing with when they are added (about 75% for models 2 and 3)."

Results of dominance analyses show that the most important variables were: Cognitive outcome- 1st parent's employment status; 2nd social emotional behavioural problems at age 3; 3rd IMD; 4th smoking in pregnancy; Physical outcome- 1st BMI pre-pregnancy; 2nd IMD; 3rd alcohol consumption in pregnancy; 4th Child sex; Behavioural outcome- 1st smoking in pregnancy; 2nd concerns about child speech; 3rd maternal age at birth; 4th parent's employment status.

AUC for three predictive models of language disability, overweight/obesity and socioemotional behavioural problems at age 11 -without prior outcome at age 3 years (complete cases, N=3,798)

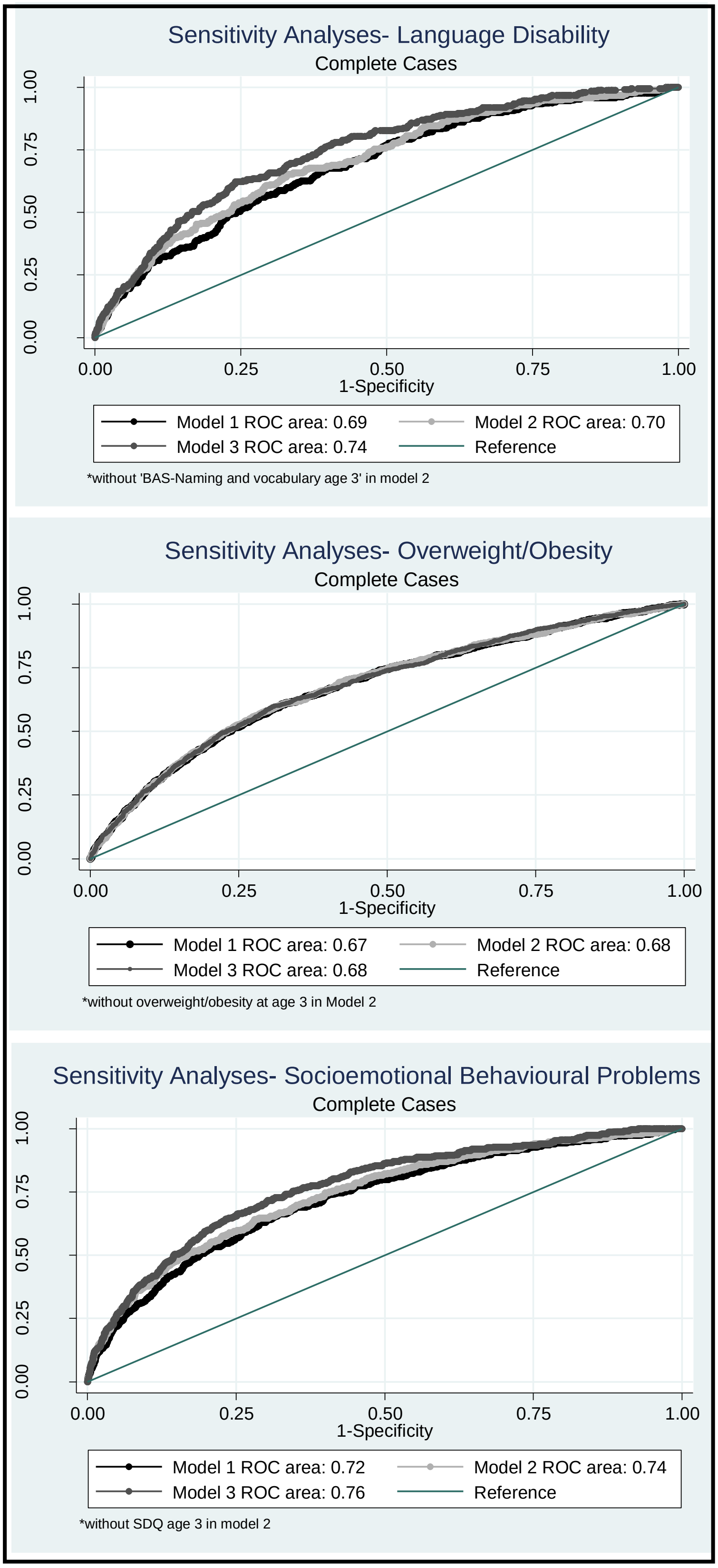

Supplement: ementary file 1 [file jech-2018-211028supp001.pdf]
